# Lambda Interferons: New Cytokines with Old Functions

**DOI:** 10.3390/ph3040795

**Published:** 2010-03-25

**Authors:** Ole J. Hamming, Hans Henrik Gad, Søren Paludan, Rune Hartmann

**Affiliations:** 1 Centre for Structural Biology, Department of Molecular Biology, Aarhus University, Aarhus, Denmark; E-Mails: ojh@mb.au.dk (O.J.H.); hhg@mb.au.dk (H.H.G.); 2 Department of Medical Microbiology and Immunology, Aarhus University, Aarhus, Denmark; E-Mail: srp@microbiology.au.dk (S.P.)

**Keywords:** Interferon, structure, innate immunity, adaptive immunity, antiviral activity, ISGF3, IFN-λR1

## Abstract

Interferon lambda (IFN-λ) is a member of the class II cytokine family, and like the other members of this family, they are small helical proteins. Since their discovery significant efforts have been made to determine their role in innate and adaptive immunity. Their strong antiviral activity, both *in vitro* and *in vivo*, has firmly established their interferon status. However, in contrast to type I interferon, only a very limited subset of cells/tissues responds to interferon lambda. In addition to inducing an antiviral state in responsive cells, recent data suggest that IFN-λ plays a role in shaping the adaptive immune response. However, the data is not in complete agreement regarding the effect of IFN-λ on the adaptive immune system. Recently IFN-λ has entered clinical trials against hepatitis C Virus and IFN-λ is a promising future therapeutic, against different viruses replicating in responsive tissues, like that of the airway epithelia. In this review we describe the knowledge acquired during the past six years about the structure and function of interferon lambda.

## 1. Introduction

Interferon lambda (IFN-λ) often referred to as the type III interferons (IFN) are a recently discovered group of small helical cytokines capable of inducing an antiviral state in responsive cells both *in vitro* as well as *in vivo*. They were discovered independently in 2003 by the groups of Sheppard [1] and Kotenko [2]. Sheppard and co-workers initially adopted an interleukin (IL-28 and 29) nomenclature based upon a phylogenetic argument, whereas Kotenko and co-workers used the IFN-λ nomenclature referring to the strong antiviral activity of these cytokines. The IFN-λ nomenclature is now the recommended nomenclature. The human genome contains three subtypes of IFN-λ; IFN-λ1 (IL-29), -λ2 (IL-28A) and -λ3 (IL-28B) encoded by 3 different genes located on chromosome 19 [1,2]. At the amino acid level IFN-λ2 and -λ3 are highly similar having 96% sequence identity while IFN-λ1 shares approximately 81 % sequence identity with IFN-λ2 and -λ3. The discovery of type III IFNs challenged our understanding of the IFN system in several ways. The receptor is composed of the IFN-λR1 and the shared IL-10R2 receptor chains, thus having a closer relationship with the interleukin-10 (IL-10) family than with the type I IFNs. Nevertheless, activation of this receptor complex by IFN-λ induces the transcription of much the same genes as do activation of the type I IFN receptor [3,4,5]. Furthermore, the genes of IFN-λ contain four introns and the genetic structure resembles that of IL-10, in contrast to the intron less genes of the type I IFN family. Finally, only a limited subset of cells and tissues responds to type III IFN, in contrast to the almost universal response to type I IFN [6,7]. 

The discovery of the type III IFNs prompted both an interest in the clinical use of IFN-λ and an active basic research field leading to significant progress over the last six years, which is the topic of this review. As our knowledge of the IFN-λ system increases, it has become evident that IFN-λ has substantial therapeutic potential. This is largely because of the favorable side effect profile compared to type I IFN. Zymogenetic, in cooperation with Bristol-Myers, has completed phase I trials and are currently undertaking phase II trials for the use of IFN-λ against hepatitis C Virus (HCV) infections. The outcome of this trial is going to be highly interesting and we hope that IFN-λ is going to be tested against other viruses replicating in IFN-λ responsive tissues, such as highly pathogenic versions of Influenza A. Nevertheless, the clinical potential of IFN-λ is, unfortunately, both beyond the scope of this review as well as beyond the competence of the authors.

## 2. Regulation of Type III IFN Synthesis

Viral infection leads to activation of Interferon Regulatory Factor 3 (IRF3), IRF7 (if present in the infected cell) as well as nuclear factor kappa B (NFκB). These transcription factors are crucial for the regulation of type I IFN genes. Binding of IRF3 and IRF7 to the promoter of IFN-λ1 leading to its induction has been demonstrated [8,9] Furthermore, activation of IRF7 induces the production of IFN-λ2 and -λ3 [9]. Binding sites for NFκB is also found in the type III IFN promoters, however, the precise role for NFκB in the activation of type III IFN genes is not well documented at the time. Thus, currently type III IFN gene regulation does not look to different from type I IFN induction. However, there is a risk that we are biased by our extensive knowledge about type I IFN gene regulation and primarily are testing pathways known to be involved in type I IFN production. Recently, a subtype of Natural Killer (NK) cells was identified which specifically produces IL-22 [10]. It will be interesting to see if a specific stimuli or a subset of cells will be identified that preferentially synthesize type III IFN, even if current knowledge speaks against this. 

## 3. IFN-λ Receptor Function and Signaling

**Figure 1:**
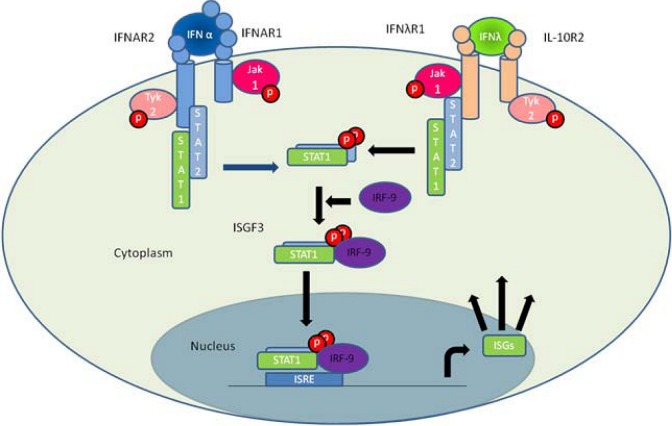
The main pathway of type I and type III interferon induced gene expression. Binding of IFN-α to the type I interferon receptor (IFNAR1 and IFNAR2) as well as IFN-λ binding to the type III interferon receptor complex (IFN-λR1 and IL-10R2) allows the JAK kinases JAK1 and TYK2 to cross phosphorylate one another. This activates the kinases leading to phosphorylation of STAT1 and STAT2 that form a STAT1-STAT2 heterodimer. The dimer binds IRF9 forming the ISGF3 complex that migrates to the nucleus where it binds to ISRE elements thus facilitating the transcription of ISGs.

IFN-λ signals *via* a heterodimeric receptor complex consisting of IFN-λR1 and IL-10R2 [2], see figure 1. IFN-λR1 is exclusively used by type III IFNs whereas IL-10R2 is shared with other members of the IL-10 family, namely IL-10, IL-22 and IL-26 [11]. The different cytokines elicit highly diverse biological responses indicating that the specificity resides within the unique receptor chain. IFN-λ induced genes were determined by gene array studies, by several groups and using different cell lines. This showed that all type III IFN induced genes were also induced by type I IFN [3,4,5]. A limited number of genes were induced preferentially by type I IFN, possibly *via* Signal Transducer and Activator of Transcription 1 (STAT1) homodimers but this could also be a result of differences in the concentration of the two cytokines. Detailed mechanistic studies revealed that stimulation of cells with either type I IFN or type III IFN results in activation of the same transcription factor, which is a complex of STAT1, STAT2 and IRF9, known as Interferon Stimulated Gene Factor 3 (ISGF3) [3]. Thus, despite using distinct receptor complexes, type I and III IFN signaling converge at ISGF3.

Both IFN-λ receptor chains consist of an extracellular, a transmembrane and an intracellular part. The extracellular part has two type III fibronectine domains [2]. Each of the type III fibronectine domains consist of around 100 aa that form two antiparallel β-sheets making a sandwich consisting of approximately seven strands. The two fibronectine domains are connected by a short linker of 5–10 aa allowing for flexibility between the two domains [12,13]. The transmembrane part is a highly lipophilic stretch of about 20–25 aa predicted to form an α-helix [2]. The IFN-λR1 receptor chain has an intracellular domain of approximately 270 aa whereas the intracellular domain of IL-10R2 is only 82 aa. The IFN-λR1 is assumed to provide the majority of binding energy but interaction with the IL-10R2 is required for proper signal transduction [14]. It is currently not clear if the IFN-λ receptor is found as a pre-associated heterodimeric complex at the surface of responsive cells or if binding of IFN-λ to IFN-λR1 leads to subsequent recruitment of IL-10R2 and formation of the ternary complex. 

The intracellular domains of the type II cytokine receptor family are known to bind members of the Janus Kinase (JAK) family. It has been shown that JAK1 is essential for IFN-λ signaling [15]. It is furthermore well established that IL-10R2 binds TYK2 [16]. Thus, binding of IFN-λ to the receptor complex activates JAK1 and TYK2 and the two kinases cross-phosphorylate and thus activate one another [17]. This leads to phosphorylation of three tyrosine residues on the intracellular part of IFNλ-R1: Tyr343, Tyr406, and Tyr517. Of these, especially Tyr343 and Tyr517 seem to be important for antiviral activity by creating a docking site for the Src Homology 2 (SH2) domain of the transcription factor STAT2 [18]. Binding of STAT proteins to IFN-λR1 brings them close to the activated JAK1 and TYK2 allowing these to phosphorylate a tyrosine residue towards the C-terminal end of the STAT proteins. These now serve as docking sites for the SH2 domains of the STAT proteins allowing for formation of STAT homo- and heterodimers [19]. In addition to activating STATs 1 and 2, which joins with IRF9 in forming ISGF3, activation of the IFN-λ receptor complex also leads to activation of STATs 3, and 5. The ISGF3 complex appears to be the main driving force of IFN-λ gene activation [3]. A correlation between STAT2 phosphorylation and antiviral activity has also been observed confirming the key role of ISGF3 in IFN-λ signaling [14]. IFN-λR1 dependent activation of both STAT3 and STAT5 was observed in BW5147 cells [18]. However, the biological consequences of STAT3 and STAT5 activation by IFN-λ are currently unclear. Furthermore, it will be interesting to see if IFN-λ induced signaling can influence post-transcriptional gene expression, especially translation of antiviral genes. 

A major difference between the type I and type III IFN systems is the receptor distribution. While the receptor for type I interferon is ubiquitously expressed, the IFN-λR1 component of the type III interferon receptor is only present on a distinct subset of cells [3,6]. Particularly cells of epithelial origin, keratinocytes as well as differentiated dendritic cells express high levels of IFN-λR1 [7,20,21,22]. The narrow distribution of IFN-λR1 means that frequent activation of this system is better tolerated by the organism as only certain specific tissues will respond. A recent study showed that human blood immune cells synthesize an IFN-λR1 variant lacking the intracellular and transmembrane parts (sIFN-λR1) *via* alternative splicing. This receptor act as a decoy by binding IFN-λ and act as a repressor of IFN-λ signaling *in vitro*. The authors argue that sIFN-λR1 is responsible for the absence of IFN-λ response in immune cells. However, when the authors wash the cells carefully to remove any soluble factors, these cells remains un-responsive to IFN-λ [22]. Data obtained in cell culture indicate that transfection of unresponsive HT1080 cells with the IFN-λR1 receptor is sufficient to render them responsive [3]. We believe that the IFN-λ system has evolved to target cells which suffer a particularly high risk of viral infection thus providing a first line of defense. However, at present we do not have sufficient experimental data to confirm this hypothesis. 

## 4. Genomic Organization

The type III IFNs have approximately 11–13% and 15–19% amino acid sequence homology with the IL-10 and type I IFN families, respectively. The IFN-λ genes are all found on the human chromosome 19q13 and on mouse chromosome 7A3 [23]. In addition to the three functional genes the human genome contains an additional pseudogene (IFN-λ4ψ). In mice the IFN-λ1 gene is also a pseudogene. The genes of type III IFNs all contain four introns and five exons. The introns are also present at equivalent positions in the IL-10 family members. In contrast to this the type I IFNs have no introns [1,24]. The IFN-λR1 gene is located on the human chromosome 1Q36 next to the IL-22R1 gene. 

## 5. Structure of IFN-λ in Relation to Other Class II Cytokines 

**Figure 2:**
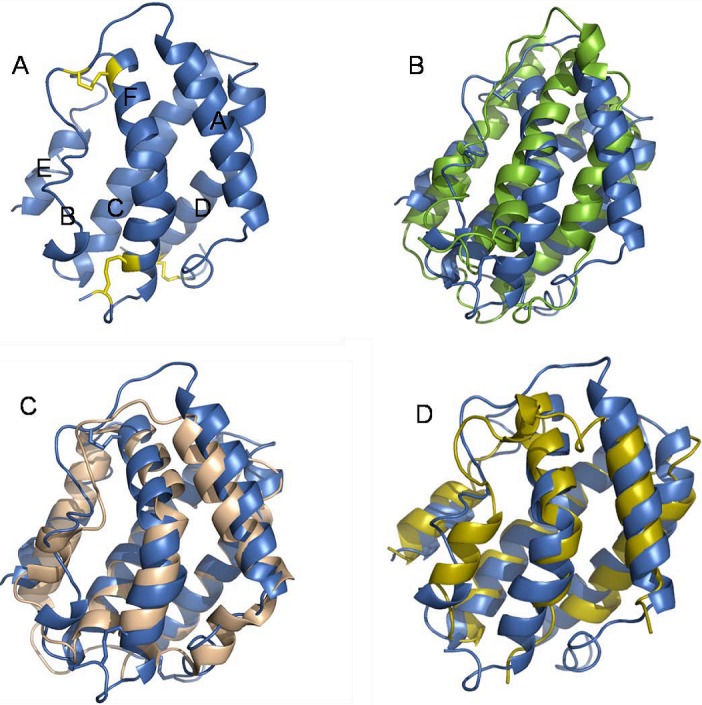
Structural comparison of IFN-λ3 to other members of the type II cytokine family. A: Cartoon representation of IFN-λ3 (PDB entry code: 3HHC). The cysteines forming the disulfide bonds are shown in yellow. The six structural elements (A-F) are indicated. B: Superposition of IFN-λ3 (blue) and IFN-α2 (PDB entry code: 1RH2) (green). C: Superposition of IFN-λ3 (blue) and IL-22 (PDB entry code: 1M4R) (light brown). D: Superposition of IFN-λ3 (blue) and IFN-γ (Created in silico from PDB entry code: 1FYH) (yellow).

In general class II cytokines consist of six distinct structural elements (A through F) connected by loops of varying length [25]. In IFN-λ, the A, C, D, E and F elements form α-helices whereas B is less ordered (Figure 2A). Helices A, C, D and F form a four helical bundle which constitutes the core of the structure. In the IFN-λ structure, helix E is relatively short and placed between element B and helix C. Helix F distinguishes type I IFN from the remaining members of the class II cytokines. In type I IFN, this helix is straight whereas it contains a characteristic bend in the 3-D structure of IL-10, IL-19, IL-22 and IFN-γ [13,26,27,28,29,30]. Two of the class II cytokines, IL-10 and IFN-γ, form intertwined dimers whereas the remaining cytokines are all monomers. The structure of IFN-λ3 was recently solved revealing a monomeric structure with a bend in helix F. Structural comparison of IFN-λ to the other members of the class II cytokines for which structures are available, showed a close structural relationship between IL-22 and IFN-λ [14] (Figure 2C). Furthermore, comparison of IFN-λ to a monomeric version of IFN-γ (created in silico) revealed substantial structural similarity (Figure 2D). Surprisingly, the structural similarity to type I IFN was limited (Figure 2B). Type I and type III IFNs are roughly the same size and have the same composition of secondary structural elements, but this is as far as the similarity goes. The difference between the strait helix F found in type I IFNs and the bending helix found in IFN-λ is obvious and furthermore the relative orientation of the remaining structural elements is rather different. 

We used a structure based mutational approach to determine the receptor binding region of human IFN-λ3. Our results indicate that helices A and F form the major site of interaction with IFN-λR1. We assume that the IL-10R2 chain binds to helices A and D at an area adjacent to the IFN-λR1 binding region [14]. However, the antiviral assay we used for determination of the specific activity of the various IFN-λ3 mutants does not allow for a strict differentiation between binding to the IFN-λR1 and IL-10R2 chains.

**Figure 3:**
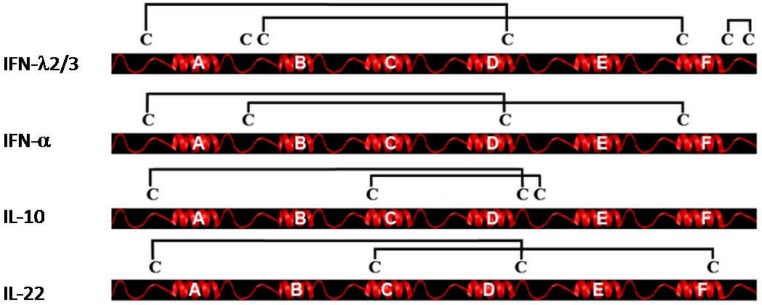
Disulfide bonding pattern of a selection of class II cytokines. The disulfide bonding pattern of IFN-λ2/3, IFN-α, IL-10 and IL-22 are shown. IFN-λ2/3 have three disulfides. The first one connects the N-terminal to the end of helix D. This bond is seen in all four type II cytokines shown. A second bond connects the AB-loop to the beginning of helix F. This bond is also seen in IFN-α. Finally IFNλ-2/3 have a third short-range bridge in the C-terminal part of the molecule. This disulfide bond is unique to IFN-λ2/3. IL-10 and IL-22 both posses disulfide bridges not seen in IFN-λ2/3. IL-10 has a bond linking helix C to the DE-loop and IL-22 has a bond connecting the N-terminal part of helix C to the end of helix F.

Another important aspect of class II cytokine structure is the pattern of disulfide bonds. In this aspect, the type III IFNs exhibit a unique pattern (Figure 3). IFN-λ1 has only five cysteines whereas IFN-λ2 and -λ3 have seven. The structure of IFN-λ3 shows three disulfide bonds; one bond connecting the N-terminal region to the end of helix D, another bond connecting the AB-loop to the beginning of helix F and the final bond forming a small loop at the C-terminal end of the protein. The first two bonds are likely to be present in IFN-λ1 as well, whereas the last bond is unique to IFN-λ2 and -3. We believe that the third disulphide bond in IFN-λ2 and -3 is of limited functional importance since it is lacking in IFN-λ1 but currently there is not sufficient data to make a firm conclusion. The disulfide bond connecting the N-terminal region to the end of helix D appears conserved in all class II cytokines except IFN-γ which has no cysteines in the mature protein as well as IFN-β and IFN-ε which are lacking the first disulfide bridge otherwise found in type I IFNs but contain the second. The second disulfide bridge connecting the AB-loop to helix F is found in both type I and III IFN but not in the other class II cytokines. Notably, this disulfide bond would prevent the formation of the intertwined dimers seen in IL-10 and IFN-γ. 

The structural similarities as well as the use of the IL-10R2 receptor chain and the gene structure suggest that IFN-λ should be placed within the IL-10 family. In addition, we have observed a significant structural similarity to IFN-γ. However, functionally it is undoubtedly an IFN and the disulfide pattern is more like type I IFN than the IL-10 family. A recent study by Fox *et al.* [31] suggested a model for interferon evolution, where both type I and III interferon originate from a single ancestral gene, which had a intron exon structure similar to that known for IFN-λ. However, this model does not explain the rather large divergence seen in type I and III IFN receptors, and how this is obtained. Thus the precise evolutionary relationship of type I and III IFN remains unclear at present. 

## 5. Potency of Different IFN-λ Subtypes

Commercially available protein preparations of IFN-λ1 and -2 were tested by several groups for their potency in an antiviral assay [6,32]. A protocol for *E. coli* expression and subsequent *in vitro* refolding of the IFN-λ protein was established for all tree human isoforms. This procedure produced protein of a higher potency than the commercially available preparations at that time. All tree human isoforms were tested and the potency of IFN-λ3 was found to be slightly higher than that found for IFN-λ1 (two fold higher as determined by EC_50_ values), however, IFN-λ2 demonstrated a significantly lower potency (16 fold lower) [33]. This finding was surprising since IFN-λ2 and -3 differ by only 6 amino acids within the mature proteins. It is currently unclear which of the amino acid differences between IFN-λ2 and -λ3 is responsible for the loss of activity.

Direct comparison of the potency of type I and type III IFN is difficult for several reasons. First, since multiple subtypes of both type I and type III IFN exist, one has to decide which subtypes to compare. Secondly, depending upon the cell line chosen one can get relatively different answers. However, if we are generalizing the current available literature it appears that type III IFN is in general weaker by at least a factor 10 as measured by EC_50_ values [33]. *In vivo* the effect of type III IFN depends upon the viral model chosen. If using a model where the virus is replicating in epithelial cells, such as vaginal herpes virus 1 infection or pulmonary influenza virus infection, the effect of type III IFN is equal to or better than type I IFN [32]. 

## 6. Role of IFN-λ *in Vivo*: Lessons from Animal Models and Studies on Human Diseases 

The first demonstration of an antiviral function of IFN-λ *in vivo* was provided by studies using a recombinant vaccinia virus expressing IFN-λ. This virus was found to be avirulent *in vivo* after intranasal infection [34]. Subsequent studies have shown that local administration of recombinant IFN-λ to mice potently inhibited replication of herpes simplex virus (HSV) and influenza A virus (IAV) in the vagina and lungs, respectively [20,32]. Interestingly, the antiviral activity of IFN-λ *in vivo* seems to be related to the site of the infection. While IAV was potently inhibited by IFN-λ in the airways, the cytokine was unable to restrict the replication of a hepatotropic IAV strain [20]. In contrast to the ubiquitous expression of the type I IFN receptor complex, it turns out that IFN-λR1 is expressed only in a narrow range of cell types [2,3,23], and that particularly epithelial cells and keratinocytes are responsive to IFN-λ treatment in the murine model [23,35]. Sommereyns and associates demonstrated pronounced expression of IFN-λR1 in the stomach, intestines, lungs and skin [7]. Furthermore they showed that in the IFN-λ-responding tissues, the epithelial cells were largely responsible for the response. Data from humans support this idea, since decreased expression of IFN-λ in the airways is associated with rhinovirus-induced asthma exacerbation [36]. Thus, type III IFNs are potent antiviral cytokines *in vivo* and target epithelial cells to induce antiviral activity.

The role of type III IFN in defense against viral infections has been addressed through the use of IFN-λR1^-/-^ mice and more recently IFN-λR1^-/-^, IFNAR^-/- ^(deficient in the type I IFN receptor complex) mice [20,35]. The only essential role for IFN-λ in antiviral defense was observed when agonists for toll-like receptors were used as topical microbicides where IFN-λR1^-/-^ mice failed to protect against a subsequent vaginal HSV infection [35]. However, when the effect of IFN-λR1 deletion was tested on an IFNAR-deficient background, It was found that whereas both the IFNAR^-/-^ and IFN-λR1^-/-^ mice displayed only marginally elevated viral titers in the lungs, the IFNAR/ IFN-λR1-double-deficient mice were highly compromised in their antiviral response, and were unable to control the virus and hence succumbed to infection, even with attenuated IAV strains [20]. These data suggest that IFN-λs do indeed contribute to innate defense against some viral infections. Probably they act in parallel with type I IFNs to mount the first line of antiviral defense in epithelial tissues, which often suffer high risk of viral infection. One should note an important difference between humans and mice in the *in vivo* biology of the type III IFN system. In humans, the IFN-λR1 chain is expressed in hepatocytes, which are responsive to IFN-λ [5,37], whereas the murine liver seems not to respond to IFN-λ treatment[20]. However, the data from the murine model do indeed suggest that IFN-λs contribute to innate defense against some viral infections. Probably they act in parallel with type I IFNs to mount the first line of antiviral defense in epithelial tissues, which often suffer high risk of viral infection. 

Further evidence for the importance of IFN-λ came from genetic studies in humans. Recent work demonstrated that genetic variation within the IFN-λ3 gene determines the likelihood of successful treatment of HCV with type I IFN [38]. Initially Ge *et al.* [38] found a polymorphism 3 kb upstream of the IFN-λ3 gene. Further sequencing of the IFN-λ3 gene in different individuals revealed two polymorphisms, a G to C transition 37 nucleotides upstream of the start codon and one non-synonymous coding single nucleotide polymorphism resulting in the K70R substitution within the IFN-λ3 protein. The K70R polymorphism resides within the AB loop of the IFN-λ structure, and the position is variable between the three human isoforms (S in λ1, R in λ2 and K in λ3). It was not possible to distinguish the three different polymorphic sites in the genetic linkage study, and thus not possible to ascribe their relative functional importance. However, functional characterization of the K70R mutation is underway. 

A second study by Thomas *et al.* [39] determined that the same genetic polymorphism as described above was also involved in determining the likelihood that patients would spontaneously clear the HCV infection [39]. The same allele which predicted high success of treatment also gave a higher likelihood of spontaneous clearance of HCV infection. These studies clearly highlight the important role played by IFN-λ in protective immunity against HCV infection and the interesting finding that the natural type III IFN system plays a significant role in the outcome of type I IFN therapy is thought-provoking. 

The role of IFN-λ in antiviral immunity is supported by the description of the secreted Y136 protein of Yaba-like disease poxvirus, which causes vesicular skin lesions in primates and can be transmitted to humans [40]. Y136 binds both type I and type III IFNs and inhibits their biological activities [40]. Altogether, IFN-λ restricts viral replication *in vivo* and seems to have a specialized role in innate immunity on epithelial surfaces often exhibiting a subdominant role in the antiviral response as compared to the type I IFN system.

## 7. IFN-λ in Adaptive Immunity

In addition to its role in innate immunity, IFN-λ is also capable of modulating the adaptive immune response. Among the genes induced by IFN-λ is MHC class I allowing infected cells to present antigens to professional immune cells thus eliciting an adaptive immune response. 

The majority of leucocytes do not respond to IFN-λ, however, during differentiation Dendritic Cells (DCs) start expressing the IFN-λR1 receptor and thus respond to IFN-λ. Type III IFN stimulated DCs are CD80 and CD40 low but have increased levels of MHC Class I and II as well as the chemokine receptor CCR7 important for migration of DCs. The IFN-λ1 stimulated DCs migrate to lymph nodes and the spleen were they efficiently induce regulatory Foxp3^+^CD25^+^CD4^+^T-cell proliferation[21]. These regulatory T-cells are known to be able to repress T-cell proliferation which is important for normal immunohomeostasis and negative regulation of the immune response [41]. These findings are in conflict with recent results from mice showing that the administration of a IFN-λ3 expressing plasmid as an adjuvant leads to lower levels of Foxp3^+^CD25^+^CD4^+^T-cells in the spleen [42]. Further studies are need to clarify whether this reflects differences in the experimental setups, differences between IFN-λ1 and IFN-λ3 or between humans and mice. 

Treatment of Naive T-cells with IFN-λ has been shown to alter the Th1/Th2 balance in favor of Th1 cells [42,43]. This is achieved by inhibiting Th2 development as seen by reduced IL-13 production and to a lesser extent by promoting Th1 development indicated by elevated IFN-γ levels [43,44]. The Th2 response is associated with high antibody titers whereas the Th1 response is characterized by strong cell mediated immunity often leading to cell death [45]. The favoring of the Th1 response and the increase of MHC class I following IFN-λ stimulation harmonizes well. Infected cells expressing MHC class I bound to antigens will be recognized and eliminated due to the strengthening of cell mediated immunity. 

Type III IFNs have also been connected with asthma. Rhinovirus infection in asthmatics generally has an elevated tendency to course asthma exacerbation if the production of IFN-λ is low [36]. This could be due to the inhibition of Th2 development following IFN-λ treatment as elevated levels of Th2 cytokines are known to be associated with asthma [36]. This is well in line with experiments showing that type III IFNs are the main IFNs produced in Bronchial Epithelial Cells (BECs) *in vivo* during Rhinovirus infection [46]. 

## 8. Anticancer Effect of Type III Interferon

Type I interferon have been approved for Cancer treatment against for instance hairy cell leukemia, malignant melanoma, and AIDS related Kaposi’s sarcoma. The side effects of Type I interferon treatment are however severe with flu like symptoms, hematopoietic toxicity anorexia and depression among others [47,48,49]. The shared gene induction profile of Type I and Type III interferon has led to investigations of the potential role of type III interferon in cancer therapy. The hope is that type III interferon will be effective against responsive cancer cells while showing fewer side effects than type I interferon due to the restricted distribution of the receptor. 

The antiproliferative and apoptotic effect of type III interferon have been shown in vitro [50,51]. Furthermore studies in mice have demonstrated the antitumor potential of type III interferon. Lasfar *et al.* [23] showed that the growth of a B16 melanoma cell line expressing murine IFN-λ2 (mIFN-λ2) was severely inhibited *in vivo* but not *in vitro* compared with B16 melanoma cells without expression of mIFN-λ2*.* The authors show an elevated expression of MHC I on the B16 melanoma expressing mIFN-λ2 compared with the regular B16 melanoma. This suggests the mobilization of a T cell based cytotoxic response against the tumor. Sato *et al*. [52] demonstrated antitumor activity of mIFN-λ against B16 melanoma and Colon26 colon cancer. First they introduced the tumor cells to the mice. After three days they injected a plasmid expressing mIFN-λ. After 30 days the mIFN-λ treated mice had smaller tumors than untreated mice. They show that this is due to apoptosis of the tumor cells as wells as increased NK activity. 

The favorable side effect profile of Type III interferon over type I interferon was recently demonstrated in a phase I study of the effect of Type III interferon against HCV. While some of the patients treated with type I interferon developed neutropenia or anemia, the patients treated with type III interferon remained symptom free [48].The data so fare shows that Type III interferon can play a positive role in cancer therapy with few or no side effects. 

## 9. Summery

Type III interferons are an interesting new addition to the interferon family, they undoubtedly induce a response in cells which is highly similar to that of type I IFN. Nevertheless, they share several structural features with the IL-10 family, including the use of the IL-10R2 receptor. It is now clear that only a limited subset of cells respond to IFN-λ, however, the precise definition of this subset is not yet completed. As tissues which experience high frequencies of viral infections seem to respond well to IFN-λ it is tempting to view this new cytokine family as a first line of defense against viral infections. It is a promising drug candidate for use against various viral infections, particularly if early observation of a very favorable side effect profile is confirmed by full scale clinical trials. However, it is still early days and it is unlikely that we have a complete picture of the actions of the type III IFN system at present. 
